# FDA Approval of Orphan Drug Indications for Pediatric Patients, 2011-2023

**DOI:** 10.1001/jamapediatrics.2024.5280

**Published:** 2024-12-09

**Authors:** Apoorva Kakkilaya, Mahnum Shahzad, Florence T. Bourgeois

**Affiliations:** 1 John Sealy School of Medicine, University of Texas Medical Branch, Galveston; 2 Department of Population Medicine, Harvard Medical School, Harvard Pilgrim Health Care Institute, Boston, Massachusetts; 3 Harvard-MIT Center for Regulatory Science, Harvard Medical School, Boston, Massachusetts; 4 Department of Pediatrics, Harvard Medical School, Boston, Massachusetts; 5 Pediatric Therapeutics and Regulatory Science Initiative, Computational Health Informatics Program, Boston Children’s Hospital, Boston, Massachusetts

## Abstract

This cohort study assesses Pediatric Research Equity Act amendments that may influence approval of orphan drug indications for pediatric patients under current regulatory programs.

Historically, many drugs have not been developed or approved for pediatric patients, resulting in high rates of off-label use.^1^ Recognizing the lack of adequate incentives for pediatric drug development, the US Food and Drug Administration (FDA) has expanded pediatric drug labeling through a series of regulatory programs. The most widely used is the Pediatric Research Equity Act (PREA), which authorizes the FDA to require sponsors to perform pediatric studies for certain indications approved for adults.^2^

Drug treatment for rare diseases is particularly relevant to pediatric populations, as many of these diseases manifest in childhood. However, except for certain oncology products, drugs designated by the FDA to treat orphan conditions are exempt from PREA study requirements. Removal of this exemption has been proposed to ensure that all relevant drugs for rare diseases are studied in children.^3,4^ To assess the potential impact of such an amendment, we examined the approval of orphan and nonorphan drug indications for pediatric patients under current regulatory programs.

## Methods

We identified all new drugs approved by the FDA’s Center for Drug Evaluation and Research from January 1, 2011, through December 31, 2023, using publicly available data.^5^ As data are deidentified and publicly available, local ethics review and informed consent were not required in accordance with the Common Rule. This cohort study followed the STROBE reporting guideline.

For each drug, we extracted information on indications approved during the study period and reviewed regulatory documents to determine orphan status and approval for pediatric patients (<18 years) at any time during the study period. For pediatric indications, we determined whether any of 3 pediatric-specific regulatory programs had been used to obtain pediatric approval (eMethods in Supplement 1).

We examined the annual percentage of indications approved with pediatric labeling among all indications approved in a given year and tested for trends using linear regression models. A 2-sided *P* < .05 by Fisher exact test was considered significant. The analysis was performed using Stata/MP, version 18 (StataCorp LLC).

## Results

The FDA approved 918 indications for 553 new drugs, of which 407 (44.3%) had orphan designations and 231 (25.2%) were labeled for pediatric use. Pediatric approval was available for 136 of 407 (33.4%) orphan indications and 95 of 511 (18.7%) nonorphan indications (*P* < .001). There was a significant increase in the annual proportion of orphan indications approved with pediatric labeling (*R*^2^ = 0.49; *P* = .003) and a positive, but nonsignificant increase in nonorphan indications approved for children (*R*^2^ = 0.03; *P* = .59) (Figure).

**Figure.**
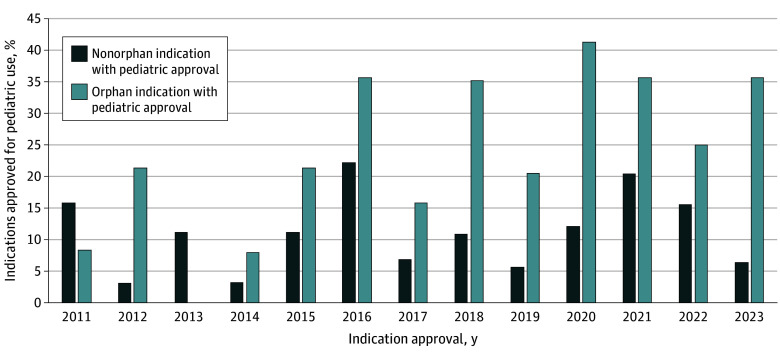
Annual Percentage of US Food and Drug Administration (FDA)–Approved Indications With Pediatric Labeling, by Orphan Designation Status Annual percentage of indications approved with pediatric labeling among all indications approved by the FDA in a given year (ie, for pediatric as well as for adult patients).

Seventy-eight pediatric orphan indications (57.4%) were approved without support of pediatric regulatory programs, whereas 81 pediatric nonorphan indications (85.3%) were supported by a pediatric program (*P* < .001) (Table). Pediatric orphan indications were more likely to include approval for younger children, with 49 orphan indications (36.1%) approved for children younger than 1 year compared with 20 nonorphan indications (21.1%) (*P* = .01).

**Table.** Indications With Labeling for Pediatric Use, 2011-2023

Variable	Indication, No. (%)			*P* value
	Total (n = 231)	Orphan (n = 136)	Nonorphan (n = 95)	
Drugs represented, No.^a^	187	116	80	NA
Timing of pediatric approval				
Original indication approval	167 (72.3)	111 (81.6)	56 (58.9)	<.001
Supplementary approval	64 (27.7)	25 (18.4)	39 (41.1)	
Pediatric regulatory program support^b^				
None	92 (39.8)	78 (57.4)	14 (14.7)	<.001^c^
PREA	78 (33.8)	7 (5.1)^d^	71 (74.7)	
BPCA	13 (5.6)	12 (8.8)	1 (1.1)	
PRV	36 (15.6)	36 (26.5)	0	
PREA and BPCA	12 (5.2)	3 (2.2)^d^	9 (9.5)	
Youngest pediatric subpopulation with approval				
Neonates (aged 0-1 mo)	35 (15.2)	24 (17.6)	11 (11.6)	.01^e^
Infants (aged 2 mo-1 y)	34 (14.7)	25 (18.4)	9 (9.5)	
Children (aged 2-11 y)	74 (32.0)	38 (27.9)	36 (37.9)	
Adolescents (aged 12-17 y)	68 (29.4)	34 (25.0)	34 (35.8)	
Age not specified	20 (8.7)	15 (11.0)	5 (5.3)	
Therapeutic area^f^^,^^g^				
Antineoplastic agents	51 (22.1)	40 (29.4)	11 (11.6)	NA
Immunomodulating agents	8 (3.5)	3 (2.2)	5 (5.3)	
Anti-infectives for systemic use	44 (19.0)	18 (13.2)	26 (27.4)	
Alimentary tract and metabolism	32 (13.9)	26 (19.1)	6 (6.3)	
Nervous system	18 (7.8)	9 (6.6)	9 (9.5)	
Dermatologics	11 (4.8)	2 (1.5)	9 (9.5)	
Various^h^	14 (6.1)	2 (1.5)	12 (12.6)	
Musculoskeletal system	10 (4.3)	10 (7.4)	0	
Antiparasitic and insecticides	10 (4.3)	7 (5.1)	3 (3.2)	
Respiratory system	8 (3.5)	4 (2.9)	4 (4.2)	
Blood and blood-forming organs	8 (3.5)	5 (3.7)	3 (3.2)	
Cardiovascular system	6 (2.6)	4 (2.9)	2 (2.1)	
Systemic hormonal preparations	6 (2.6)	5 (3.7)	1 (1.1)	
Genitourinary system and sex hormones	3 (1.3)	0	3 (3.2)	
Sensory organs	3 (1.3)	2 (1.5)	1 (1.1)	

Abbreviations: BPCA, Best Pharmaceuticals for Children Act; NA, not applicable; PREA, Pediatric Research Equity Act; PRV, Pediatric Rare Disease Priority Review Voucher.

^a^ There were 9 drugs with both nonorphan and orphan indications; therefore, the number of drugs in these 2 groups is greater than the total of 187 drugs.

^b^ A description of the pediatric regulatory programs is provided in the eMethods in Supplement 1.

^c^ Represents comparison of none vs any pediatric regulatory program.

^d^ While orphan-designated indications are exempt from PREA study requirements, certain indications received orphan designation for the pediatric subpopulation after the PREA study requirements were issued for the adult indication. In addition, the Research to Accelerate Cures and Equity Act authorizes the US Food and Drug Administration to require pediatric studies for certain oncology drugs, even if the indication has an orphan designation.

^e^ Represents comparison of patients younger than 1 year with those aged 2-17 years.

^f^ Based on Anatomical Therapeutic Chemical classification system.

^g^ There was 1 orphan drug with 2 classifications; therefore, the number of drugs in the total and orphan column is greater than the total of 231 and 136, respectively.

^h^ Includes contrast agents, a neuromuscular reversal agent, an iron chelator, and a binding agent for hyperkalemia.

## Discussion

In this comprehensive analysis of drugs approved by the FDA over 13 years, orphan indications were more likely to be approved for pediatric use than nonorphan indications, even with the exemption for PREA study requirements in place. While most orphan indications received pediatric approval without pediatric-specific regulatory programs, three-quarters of nonorphan indications were supported by one of the programs. A study limitation is the lack of information on sponsor considerations in seeking pediatric approval for different indications and the influence of pediatric regulatory programs on pediatric development plans.

The FDA has reported that as much as 36% of rare disease drugs relevant to children are approved without complete pediatric information,^6^ indicating that additional authority to require pediatric study of these drugs may be beneficial. Our findings highlight the need for further examination of potential gaps in regulatory processes for nonorphan indications and to ensure that PREA amendments maximize pediatric research across all conditions relevant to children.

## Notes

Supplement 1.
eMethods

eReferences

Supplement 2.
Data Sharing Statement
