# Supplementary material for: FDA Approval of Orphan Drug Indications for Pediatric Patients, 2011-2023
Source: JAMA Pediatr. 2024 Dec 9;179(2):203–5. doi: 10.1001/jamapediatrics.2024.5280 (PMC11791693; doi:10.1001/jamapediatrics.2024.5280)
Supplement: Supplement 1. — eMethods eReferences [file jamapediatr-e245280-s001.pdf]

Supplemental Online Content

Kakkilaya A, Shahzad M, Bourgeois FT. FDA approval of orphan drug indications for pediatric patients, 2011-2023. *JAMA Pediatr*. Published online December 9, 2024.
doi:10.1001/jamapediatrics.2024.5280

eMethods

eReferences

This supplemental material has been provided by the authors to give readers additional information about their work.

© 2024 American Medical Association. All rights reserved.

eMethods

Data Collection on Use of Pediatric-Specific Regulatory Programs

For all indications with pediatric approval, we determined whether any of three pediatric-specific regulatory programs had been used to obtain the first pediatric approval. The first of these programs was the Pediatric Research Equity Act (PREA), which authorizes the FDA to require sponsors to perform pediatric studies for certain indications approved in adults. Indications approved via PREA are described in FDA regulatory documents and the FDA's database on pediatric labeling changes.[1] The second was the Best Pharmaceuticals for Children Act (BPCA), which provides an incentive whereby sponsors receive an extension of patent exclusivity in return for voluntarily conducting specified pediatric studies. Information on use of the BPCA program was obtained from the FDA's database on pediatric exclusivity determinations.[2] A third program, the Rare Pediatric Disease Priority Review Voucher (PRV) program, aims to incentivize drug development specifically for rare pediatric diseases. Under this program, sponsors are awarded a voucher that can be used to expedite the approval of another product in return for bringing a rare pediatric disease drug to market. Information on issuance of PRVs was obtained from the U.S. Federal Register.[3]

Trends in Percentage of Indications Approved with Pediatric Labeling

While the main analysis examined pediatric labeling obtained both at the time of original indication approval and as supplementary approvals at any time during the study period, the trend analysis was limited to pediatric labeling available at the time of original indication approval. The two approval types could not be combined in the trend analysis since

© 2024 American Medical Association. All rights reserved.

supplementary approvals are not linked to the year of indication approval and there would be a time bias with older indications more likely to receive pediatric approval compared to more recently approved indications.

eReferences

1. U.S. Food and Drug Administration. Pediatric Labeling Changes. Accessed August 9, 2024. https://www.fda.gov/science-research/pediatrics/pediatric-labeling-changes

2. U.S. Food and Drug Administration. List of Determinations Including Written Request. Accessed August 9, 2024. https://www.fda.gov/drugs/development-resources/list-determinations-including-written-request

3. National Archives and Records Administration. Federal Register. Accessed August 9, 2024. National Archives and Records Administration,

© 2024 American Medical Association. All rights reserved.
