# Supplementary material for: FDA Approval of Orphan Drug Indications for Pediatric Patients, 2011-2023
Source: JAMA Pediatr. 2024 Dec 9;179(2):203–5. doi: 10.1001/jamapediatrics.2024.5280 (PMC11791693; doi:10.1001/jamapediatrics.2024.5280)
Supplement: Supplement 2. — Data Sharing Statement [file jamapediatr-e245280-s002.pdf]

Data Sharing Statement

Kakkilaya. FDA Approval of Orphan Drug Indications for Pediatric Patients, 2011-2023. *JAMA Pediatr*. Published December 09, 2024. doi:10.1001/jamapediatrics.2024.5280

Data
Data available: Yes
Data types: Other (please specify)
Additional Information: Data on FDA approval of drugs
How to access data: All data used in the analyses are publicly available.
When available: With publication

Supporting Documents
Document types: None

Additional Information
Who can access the data: Any investigator
Types of analyses: Any analysis type
Mechanisms of data availability: All data used in the analyses are publicly available.
